# New Insights into How Increases in Fertility Improve the Growth of Rice at the Seedling Stage in Red Soil Regions of Subtropical China

**DOI:** 10.1371/journal.pone.0109161

**Published:** 2014-10-07

**Authors:** Yilin Li, Weiming Shi, Xingxiang Wang

**Affiliations:** 1 State Key Laboratory of Soil and Sustainable Agriculture, Institute of Soil Science, Chinese Academy of Sciences, Nanjing, China; 2 Jiangxi Key Laboratory of Ecological Research of Red Soil, Yingtan, Jiangxi, China; Universidade Federal de Vicosa, Brazil

## Abstract

The differences in rhizosphere nitrification activities between high- and low- fertility soils appear to be related to differences in dissolved oxygen concentrations in the soil, implying a relationship to differences in the radial oxygen loss (ROL) of rice roots in these soils. A miniaturised Clark-type oxygen microelectrode system was used to determine rice root ROL and the rhizosphere oxygen profile, and rhizosphere nitrification activity was studied using a short-term nitrification activity assay. Rice planting significantly altered the oxygen cycling in the water-soil system due to rice root ROL. Although the oxygen content in control high-fertility soil (without rice plants) was lower than that in control low-fertility soil, high rice root ROL significantly improved the rhizosphere oxygen concentration in the high-fertility soil. High soil fertility improved the rice root growth and root porosity as well as rice root ROL, resulting in enhanced rhizosphere nitrification. High fertility also increased the content of nitrification-induced nitrate in the rhizosphere, resulting in enhanced ammonium uptake and assimilation in the rice. Although high ammonium pools in the high-fertility soil increased rhizosphere nitrification, rice root ROL might also contribute to rhizosphere nitrification improvement. This study provides new insights into the reasons that an increase in soil fertility may enhance the growth of rice. Our results suggest that an amendment of the fertiliser used in nutrient- and nitrification-poor paddy soils in the red soil regions of China may significantly promote rice growth and rice N nutrition.

## Introduction

Nitrification, an important component of the nitrogen cycle, involves two aerobic microbial processes. In nitrification, ammonia (NH_3_) is first converted to nitrite (NO_2_^−^); then, NO_2_^−^ is further converted to nitrate (NO_3_^−^) [1], [2]. Nitrification occurs in a variety of environments, such as sediments and soils. However, in waterlogged environments, such as paddy soils and flooding waters, nitrification is limited by the low availability of oxygen, which results in low NO_3_^−^ availability. An increased number of hydroponic experiments have demonstrated that rice absorbs and assimilates NO_3_^−^ in preference to ammonium (NH_4_^+^) [3], [4]. Rice growth, yield, net N acquisition and N use efficiency (NUE) have been shown to be superior using mixed NH_4_^+^ and NO_3_^−^ nutrition compared with pure NH_4_^+^ cultivation [3], [5], [6]. In paddy fields, NO_3_^−^ concentrations often fluctuate and are difficult to control compared with hydroponic culturing, primarily because of denitrification in the bulk soil and nitrification in the rhizosphere. In our previous soil culture study [7], different rice cultivars (Indica and Japonica) showed a significant difference in the nitrification activity in their rhizospheres, and rhizosphere nitrification was closely related to rice growth and N nutrition as well as rice N accumulation and NUE.

The oxidation of NH_3_ is a microbial process and is thought to be the rate-limiting step for nitrification [8]. Recently, according to metagenomic [9], [10] and cultivation [11] methods, another type of NH_3_ oxidiser, the ammonia-oxidising archaea (AOA) group, has been found to be abundant in the natural environment [12] and has been hypothesised to be a dominant group among the ammonia-oxidising prokaryotes in the soil [13]. Because ammonia-oxidising bacteria (AOB) and AOA are both strictly aerobic chemolithoautotrophic microorganisms, nitrification can only occur in the presence of oxygen and is restricted in the oxic upper few millimetres of flooded soils [14]–[16].

However, rice plants form numerous adventitious roots containing aerenchyma. These roots promote radial oxygen loss (ROL) from the roots to the rhizosphere, supporting aerobic microbial nitrification [17], [18]. Furthermore, ROL alleviates the phytotoxicity of reduced compounds (e.g., Fe^2+^, Mn^2+^, H_2_S) in rice [19]. ROL is of biological significance for submerged plants and has been investigated extensively using various methods [20]–[23]. A Clark-type oxygen microelectrode has been used to measure the rice ROL and the detailed oxygen vertical profiles in the field, indicating that rice plants at 21 d had 20% air saturation at the root surface. Diffusion of O_2_ away from the root was able to create oxic conditions up to a distance of 0.4 mm away from the root [23]. The diameter of this oxygen microelectrode was only 25 µm, which should have caused minimal damage to the soil and rice plants.

Two paddy soils (representing low and high fertilities) in red soil regions with low nitrification activity were examined by oxygen microelectrode techniques to study the relationship between rice ROL and rhizosphere nitrification and the contribution of root ROL to rice N nutrition. Red soils, which can be classified as acrisols and ferralsols according to the Food and Agriculture Organization (FAO) nomenclature [24], are widespread in tropical and subtropical China, covering 11.8% of the nation's land area. The red soil region contains abundant water resources and heat resources and is one of the principal rice production areas in China [25]. Due to intensive leaching and weathering, long-term inappropriate utilisation and management, and the inherent fragility of hill ecosystems, the red soils are characterised by low pH and fertility, as well as low nitrification activity [26], [27]. When natural factors, such as parent materials, topography, and climate, are the same in the paddy soil-forming process, time and human factors, such as long-term fertilisation, are the primary influencing factors in improving soil fertility. Two paddy soils collected from paddy fields with different fertility levels were defined as low fertility (i.e., reclamation of approximately 15 years, long-term inorganic and organic fertilisation in the rice season) and high fertility (i.e., reclamation great than 50 years, long-term inorganic and organic fertilisation in the rice season and manure application in the fallow winter). Although increasing soil fertility may improve rice root growth, little is known about whether it could change the rice root aerenchyma development and ROL. Our previous study showed that oxygen concentrations in paddy soil (without rice plants) with high fertility were much lower than those measured in low-fertility paddy soil collected from red soil regions (unpublished data). In this study, we hypothesised that rice plants grown in high-fertility paddy soil would improve the root aerenchyma development and ROL, which would lead to increased oxygen in the rhizosphere soil, and, thus, enhance rhizosphere nitrification activity.

## Materials and Methods

### Paddy soils and rice plant cultivars

Paddy soils derived from Quaternary red clay (hydragric anthrosols according to the FAO nomenclature [24]) were collected from the Chinese Academy of Sciences Red Soil Ecological Experimental Station, located in Yingtan (28°15′20″ N, 116°55′30″ E), Jiangxi Province, which is one of the major rice production areas in China. The cultivation practice in the area is two crops per year. The Yingtan region has a typical subtropical monsoon climate with an annual precipitation of 1785 mm and a mean annual temperature of 17.8°C. Two paddy soils (0–20 cm) collected from paddy fields with different fertility levels were defined as low fertility and high fertility, and their properties are described in Table 1. The soil samples were air-dried, ground, sieved through 0.71-mm mesh, and stored for further incubation experiments. The plant residues were carefully removed by hand. One of the most popular Indica rice cultivars in Jiangxi Province, Hesheng 10, was used in the experiment.

**Table 1:** Properties of the paddy soils used in the experiment.

Property	Low fertility	High fertility
pH (water:soil, 2.5∶1)	4.75	5.02
Organic matter (g kg^−1^)	17.5	39.1
Total N (g kg^−1^)	0.85	1.74
Total P (g kg^−1^)	0.32	0.67
NH_4_^+^-N (mg kg^−1^)	0.72	2.27
NO_3_^−^-N (mg kg^−1^)	0.17	0.21
Clay (%)	36.3	20.9
Average yield of double-cropping rice per year (kg hm^−1^)	7500	10000
Reclamation years	15	>50

### Soil incubation and sampling

A rhizobox, as described by Li *et al*. [28], was used to collect soil samples at various distances from the rice root surface. Two pieces of nylon nets (with a 30-µm mesh size) were used to divide the rhizobox into three compartments to facilitate the isolation of the rice root from the rhizosphere soil. Three rice plants were planted in the middle compartment of each rhizobox. The mesh of the nylon net was fine enough to allow the water and nutrient elements to pass through. A 600-g sample of the paddy soil (air dried and sieved) was mixed thoroughly with urea (120 mg N kg^−1^) and KH_2_PO_4_ (93 mg kg^−1^) to fill each rhizobox (the fertiliser applications were performed according to the conventional amounts used by the local farmers to support rice growth adequately). A rhizobox without rice plants was used as the control (CK). One centimetre of surface water was maintained after rice germination, using deionised water every morning and evening throughout the incubation. The rice plants were incubated at 25°C with a 16-h photoperiod during the experiment. Three replicate rhizoboxes for each treatment were used, and soil samples were collected at 40, 50 and 60 d after rice germination; an additional six replicate rhizoboxes were used for in situ measurements of soil oxygen concentration and rice root ROL 50 d after germination.

A previous experiment in this laboratory showed that between 40 and 60 days after rice germination, the rice roots were fully developed and filled the middle compartment of the rhizobox so that the nylon nets would not influence the rice root growth. Therefore, the soil adhering to the rice roots in the inner rhizobox was defined as root surface soil, and the soil in the outer rhizobox within a 4-mm distance from the nylon nets was defined as rhizosphere soil. To prevent the water layer from interfering with the soil mineral N and nitrification activity determination, no watering occurred on the evening of the day prior to sampling to maintain a thin (1–2 mm) water layer. The sampling was conducted at 10:00 am, and each treatment (three replicates) was first frozen at −20°C for 2 h to harden the paddy soil for subsequent slicing. First, the rhizobox was carefully split along the two nylon nets into three compartments: middle, right, and left parts. Next, a piece of organic glass board (2 mm thick, 8 cm×8 cm) was inserted into the inner side of the right or left part of the rhizobox. Then, a soil sample of 2-mm thickness was pushed aside, and the soil was sliced to obtain a soil sample located 2 mm away from the root surface. Next, another piece of organic glass board (2 mm thick, 8 cm×8 cm) was inserted to obtain a layer located 4 mm away from the root surface. Using the same procedure, soil samples were obtained at distances of 6, 8, 10, 20, 30 and 40 mm from the root surface. The soil samples collected in the right and left compartments at the same distance were mixed together for the assessments. The root surface soil was separated from the rice roots using sterilised tweezers. All soil samples were collected at the same time as the plant sampling to assay for mineral N and nitrification activity.

### Mineral N assays

Fresh soil samples were extracted with 2 mol L^−1^ of KCl (soil:solution ratio: 1∶10), and the extracts were measured colourimetrically for NH_4_^+^ and NO_3_^−^ [26] using a continuous flow auto- analyser (model Autoanalyzer 3, Bran + Luebbe, Hamburg, Germany).

### Short-term nitrification activity assays

Short-term nitrification estimates are typically used for the nitrification activity assay. The principle underlying this method is based on the determination of NO_2_^−^ after the incubation of soil samples with NaClO_3_ in the absence of NH_4_^+^ for 24 h at 25°C [29], [30]. Briefly, each soil sample (5 g) was shaken with 2.5 mL of NaClO_3_ (75 mmol L^−1^, which prevents oxidation of NO_2_^−^) at 170 rpm on a rotary shaker (model HZ-9611K, Hualida Laboratorial Equipment Co., Ltd., Taicang, China) for 30 min, and then incubated aerobically in an incubator for 24 h at 25°C. After incubation, NO_2_^−^ was extracted from the soil samples into a total volume of 15 mL using two solutions (5 mL of deionised H_2_O followed by 10 mL of 2 mol L^−1^ KCl) by shaking at 170-rpm for 30 min on a rotary shaker, as described previously. The contents were mixed thoroughly and immediately filtered. Five millilitres of the clear filtrate was pipetted into glass test tubes, followed by 3 mL of the buffer (0.19 mol L^−1^ NH_4_Cl, pH 8.5) and 2 mL of the reagent (1% sulphanilamide, 0.05% N-(1-naphthyl) ethylene-diamine dihydrochloride and 10% phosphoric acid mixed solution), for NO_2_^−^ determination. The contents were again vigorously shaken and allowed to stand for 15 min at room temperature. For a control measurement, soil samples were extracted as previously described after incubation with NaClO_3_ at −20°C. The amount of NO_2_^−^ produced was measured based on the absorbance at 520 nm in a spectrophotometer. Pre-experiments using the paddy soil without planted rice (waterlogged for 40 d) to observe the kinetics of ammonia oxidation in the first 24 hours were performed and revealed that nitrite was produced linearly over time (p<0.05).

### In situ measurements of soil oxygen concentrations

An additional three replicate rhizoboxes were used for the in situ measurement of soil oxygen concentrations 50 d after germination. The incubation conditions were the same as described above except that a 1-cm water layer was maintained in the rhizoboxes for the in situ measurement.

The oxygen microelectrode was a miniaturised Clark-type oxygen electrode with a guard cathode (OXY25, ø = 20–30 µm, Unisense, Aarhus, Denmark [31]). The soil oxygen profile measurements were performed in the right and left compartments of each rhizobox and in the centre of the range at a distance of 2 mm from the nylon nets for all treatments. All measurements were performed with a 50-µm depth interval using a microelectrode with a diameter of approximately 25 µm, and the periods for “wait before measure” and “measure” were both set as 3 s.

### In vitro measurements of rice root oxygen loss in an agar microcosm

Three rhizosphere boxes were split into three compartments each, and the rice plants with soil were placed into a plastic bucket filled with deionised water. The soil adhering to the rice roots was removed by repeated immersion in the deionised water. After the soil was removed, the rice roots were separated according to the individual plants and were then individually placed into beakers with International Rice Research Institute (IRRI) rice nutrient solution (pH 5.5) [32] for 2 h. The roots of each individual rice plant were then inserted into a container filled with IRRI rice nutrient solution (pH 5.5) containing 0.9% agar [23] before the agar was allowed to solidify at 38°C. The container was combined with one small culture dish (6 cm in diameter, 2.5 cm in depth) and one large culture dish (15 cm in diameter, 2.5 cm in depth). The rice root (to be measured) was inserted into the small culture dish, whereas the other roots were evenly distributed in the large culture dish. Next, paraffin oil was poured onto the surface of the solidified agar in the small culture dish to prevent air penetration. To obtain comparable results, the sample root was placed horizontally in the agar. Because the solidified agar was a clear medium, it allowed observation of the rice roots and provided fixation support for the roots. To measure the oxygen levels of the outer and inner rice roots, the microelectrode had to be sufficiently thin to longitudinally penetrate the root. Therefore, a much slimmer microelectrode (OXY10, ø = 8–10 µm, Unisense, Aarhus, Denmark [31]) was used for the measurement. Newly formed roots with lengths of approximately 45 mm were selected and measured at 25 mm from the root tip. The measurements were conducted after the rice roots had been fixed in the agar for approximately 2 h; these measurements were repeated for different rice plants with different newly formed roots with the same length as described above. All measurements were conducted with a 50-µm depth interval, and the periods for “wait before measure” and “measure” were both set to 3 s.

Because oxygen concentration is very sensitive to temperature, soil and agar temperatures were monitored and recorded during the oxygen measurements. All of the oxygen measurements, including soil oxygen concentration and root ROL, were performed in a specialised laboratory without electrical noise interference at a temperature of 20°C.

### Plant dry matter and N accumulations assays

The rice tissues were separated into shoots and roots and heated at 105°C for 30 min to terminate enzyme activity. The samples were then oven-dried at 70°C for 48 h to a constant weight. The dried plant material was ground and digested for total N determination using the Kjeldahl method. A 5-mL aliquot from the 100-mL digested solution was then analysed for N using a continuous flow auto-analyser (model Autoanalyser 3, Bran + Luebbe, Hamburg, Germany).

### Plant NRA and GSA assays

Fresh leaves and roots of both treatments were immediately frozen with liquid N and then stored in a refrigerator at −70°C for the later measurement of glutamine synthetase activity (GSA) and NO_3_^−^ reductase activity (NRA).

The level of GSA was assayed using an in vitro method. Frozen plant material (0.5 g) was ground in a precooled mortar with sand and a pestle after adding liquid N and then homogenised in an extraction buffer (pH 7.2, 10 mL g^−1^ fresh weight) containing 0.5 mmol L^−1^ of EDTA and 50 mmol L^−1^ of K_2_SO_4_. The homogenates were centrifuged at 20,000 *g* for 20 min. Next, 1.2 mL of the clear filtrate was pipetted into a centrifuge tube, to which was then added 0.6 mL of imidazole-HCl (pH 7.0, 0.25 mol L^−1^), 0.4 mL of sodium glutamate (pH 7.0, 0.3 mol L^−1^), 0.4 mL of ATP-Na (pH 7.0, 15 mmol L^−1^), 0.2 mL of MgSO_4_ (0.5 mol L^−1^) and 0.2 mL of hydroxylamine (1 mol L^−1^). After the mixture was incubated at 25°C for 20 min, the reaction was terminated by adding 0.8 mL of acidic FeCl_3_ [24% (W/V) trichloroacetic acid and 10% (W/V) FeCl_3_ in 18% HCl]. The amount of γ-glutamyl hydroxamate was measured with a spectrophotometer at 540 nm. One unit of GS activity was defined as the amount of enzyme that catalysed the formation of 1 µmol γ-glutamyl hydroxamate per min at 25°C [33].

The level of NRA was assayed by another in vitro method. Frozen plant material (0.5 g) was ground in a precooled mortar with sand and a pestle after adding liquid N and then homogenised in an extraction buffer (10 mL g^−1^ fresh weight) containing 5 mmol L^−1^ of EDTA, 5 mmol L^−1^ of cysteine and 25 mmol L^−1^ of potassium phosphate buffer (pH 8.7). The homogenates were centrifuged at 20,000 *g* for 20 min. Next, 0.4 mL of the clear filtrate was pipetted into a centrifuge tube, and the filtrate was added to 1.0 mL of KNO_3_ (0.1 mol L^−1^) and 0.6 mL of NADH (2 mg mL^−1^). After the mixture was incubated at 25°C for 30 min, the excess NADH was oxidised by the addition of 0.5 mL of sulphanilamide (1%). The mixture was centrifuged at 20,000 *g* for 15 min. The amount of NO_2_^−^ produced was measured after the addition of N-(1-naphtyl) ethylene-diamine dihydrochloride (0.02%) using an absorbance of 540 nm in a spectrophotometer [34].

### Plant POR assay

For this assay, 0.4–0.6 g of fresh adventitious roots was sampled according to published methods [13], [35], [36]. POR was determined as , where POR is the root porosity (%), P_gr_ is the mass of the pycnometer with water and ground roots (g), P_r_ is the mass of the pycnometer with water and roots (g), r is the mass of roots (g), and P is the mass of the pycnometer with water (g).

### Data analysis

All statistical analyses were performed using SPSS version 13.0. A one-way ANOVA with a homogeneity of variance test was used, followed by a least significant difference (LSD) test to check for quantitative differences between treatments. The statistically significance level was set at *p*<0.05.

## Results

### Distribution of soil nitrification activity

**Figure 1:**
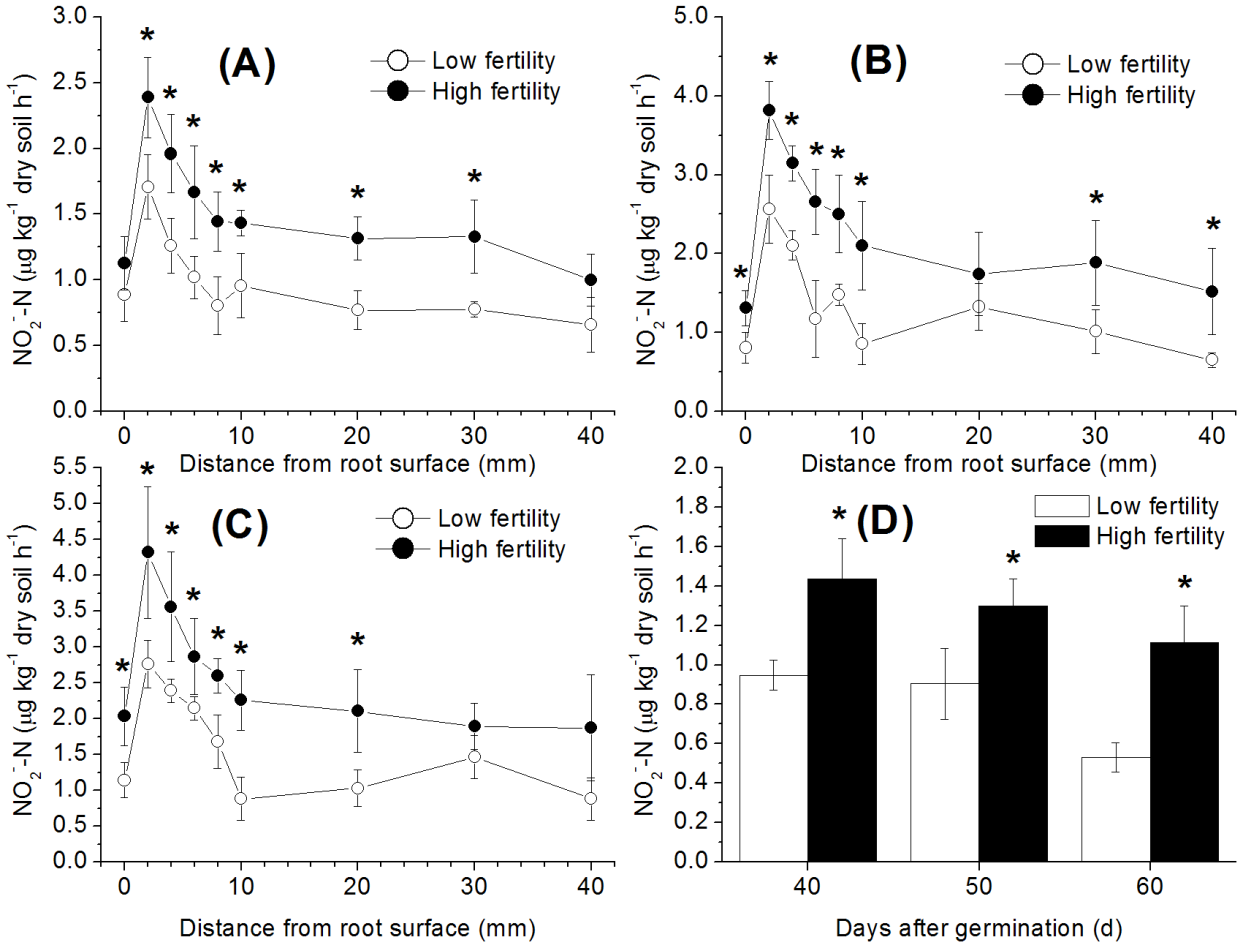
Nitrification activities measured at various distances from the rice root surface in paddy soils with different fertility levels (A) 40 d after germination, (B) 50 d after germination, (C) 60 d after germination, relative to the (D) control (without rice plants). The bars indicate ±SD. Dots and bars with * indicate a significant difference (p<0.05) between the two fertility levels.

The nitrification activities of the root surface and rhizosphere soils increased over time, whereas those in the bulk soil did not change significantly (Fig. 1A, B and C). The maximal nitrification activities were found 2 mm away from the root surface in the rhizosphere soil, and their values were 2.76 (0.33) µg kg^−1^ h^−1^ for the low-fertility treatment and 4.32 (0.92) µg kg^−1^ h^−1^ for the high-fertility treatment for the last sampling period (Fig. 1C). Minimal nitrification activities appeared in the root surface and bulk soils (20–40 mm away from the root surface). Throughout the sampling period, the nitrification activities in the paddy soil with high fertility were always significantly higher than those in the soil with low fertility, except for the root surface soil 40 d after germination (Fig. 1A). Compared with the control, rice planting generally increased the rhizosphere soil nitrification activity significantly, but no increased effect of rice planting on nitrification occurring in the root surface or bulk soil was observed in either treatment. For example, the nitrification activity 2 mm away from the root surface was 1.8, 2.8 and 5.2 times higher than that in the control in the low-fertility soils at 40, 50 and 60 d after germination, respectively, and the corresponding activity in the high-fertility soil was 1.7, 2.9 and 3.9 times higher than the control, respectively (Fig. 1A, B, C and D).

### Distribution of soil nitrate concentration

**Figure 2:**
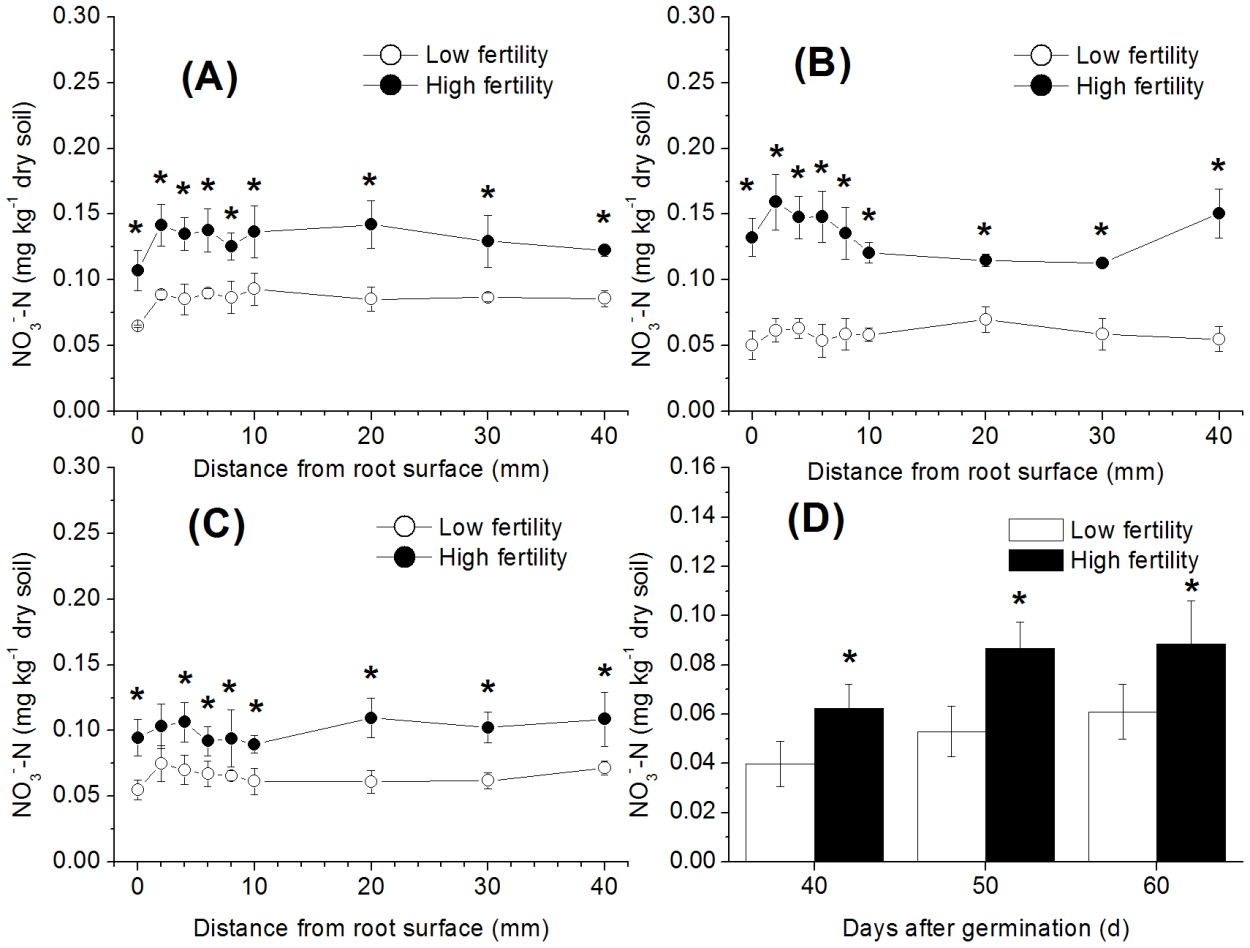
NO_3_^−^-N measured at various distances from the rice root surface in paddy soils with different fertility levels (A) 40 d after germination, (B) 50 d after germination, and (C) 60 d after germination, relative to the (D) control (without rice plants). The bars indicate ±SD. Dots and bars with * indicate a significant difference (p<0.05) between the two fertility levels.

The NO_3_^−^ concentration did not change over time. Although there was a strong absorption of NO_3_^−^ by the rice roots, the horizontal distribution of soil NO_3_^−^ at different distances from the root surface showed no concentration gradient (Fig. 2A, B and C). This result might have been due to the strong mobility of NO_3_^−^ in the soil. The NO_3_^−^ concentrations in the high-fertility soil were always higher than those in the low-fertility soil. This difference which might have contributed to the higher nitrification activity of the high-fertility soil described above (Fig. 1A, B, and C). The NO_3_^−^ concentration in the soil planted with rice did not differ significantly from that in the control (Fig. 2A, B, C and D).

### Distribution of the soil ammonium concentration

**Figure 3:**
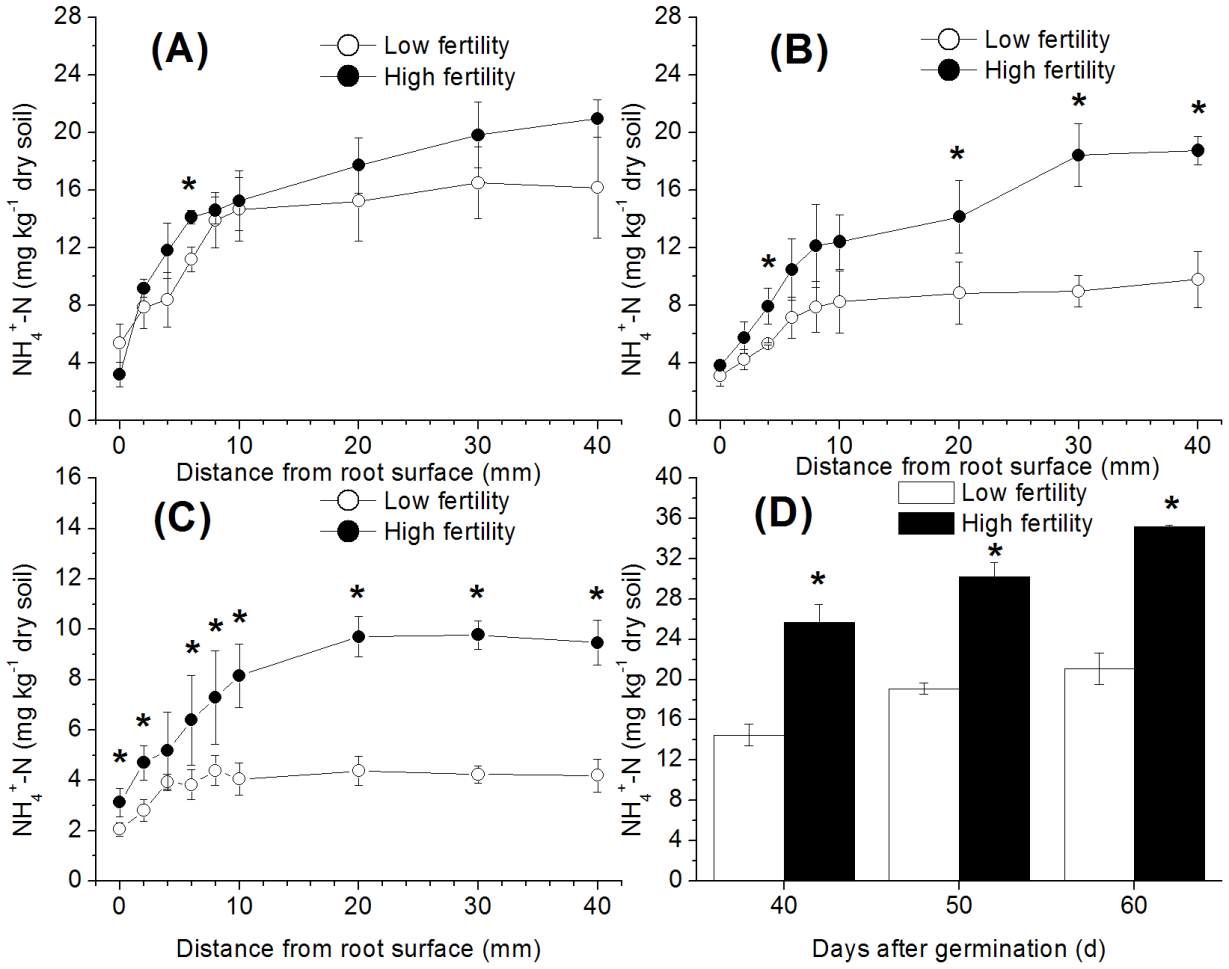
NH_4_^+^-N measured at various distances from the rice root surface in paddy soils with different fertility levels (A) 40 d after germination, (B) 50 d after germination, and (C) 60 d after germination, relative to the (D) control (without rice plants). The bars indicate ±SD. Dots and bars with * indicate a significant difference (p<0.05) between the two fertility levels.

The NH_4_^+^ concentration in the soil decreased over time (Fig. 3), suggesting that a large quantity of NH_4_^+^ was absorbed by the rice roots. This conclusion is partially supported by the distinct depletion of NH_4_^+^ in the zone near the root surface (Fig. 3A, B, and C), where the NH_4_^+^ concentration increased with increasing distance from the rice roots. In the period 40–60 d after germination, the NH_4_^+^ concentrations of the high-fertility treatment in the bulk soil were always higher than those of the low-fertility treatment, except for the measurement taken 40 d after germination (Fig. 3A). This difference increased over time (Fig. 3A, B, and C). As the NH_4_^+^ concentrations in the control increased slightly with time, the NH_4_^+^ concentrations in the control for the high-fertility treatment were significantly higher than those in the control for the low-fertility treatment (Fig. 3D). Due to the NH_4_^+^ uptake by rice roots, the content of NH_4_^+^ in the treatments with rice was significantly lower than that in the control (without rice), especially on the last sampling day. For example, the NH_4_^+^ concentrations 40 mm away from the rice root (bulk soil) were 19.9% and 26.9% of those in the control at 60 d after germination in the low- and high-fertility soils, respectively, and those in the root surface soil were only 9.75% and 8.86% of the control.

### Rice plant nitrogen assimilation, growth, and nitrogen accumulation

The NRA and GSA in the rice leaves and roots did not change over time, and the levels of NRA and GSA in the rice leaves were significantly higher than those in the roots (Table 2). In addition, high soil fertility ameliorated the NRA and GSA levels significantly; the leaf and root NRAs increased by 48.2% and 49.7% in the high-fertility soil compared to those in the low-fertility soil, whereas the leaf and root GSA levels in the high-fertility soil were, respectively, 37.6% and 103.1%, greater than those in the low-fertility soil (Table 2).

**Table 2:** Rice plant N assimilation, growth, and nitrogen accumulation^a^.

Fertility level	Sampling date (d)	NRA (µg g^−1^ FW h^−1^)		GSA (mmol g^−1^ FW min^−1^)		Biomass (g DW plant^−1^)	N accumulation (mg N plant^−1^)
		Leaf	Root	Leaf	Root		
Low fertility	40	7.21±0.93 ^b^	4.06±0.33 ^c^	1.85±0.14 ^c^	0.08±0.02 ^b^	0.49±0.10 ^d^	16.8±1.77^d^
	50	6.84±0.76 ^b^	4.61±0.82 ^bc^	2.09±0.05 ^bc^	0.09±0.01 ^b^	0.85±0.09 ^c^	23.4±0.93 ^c^
	60	7.38±0.30 ^b^	4.14±1.08 ^c^	2.35±0.15 ^bc^	0.11±0.04 ^b^	1.32±0.15 ^b^	34.8±3.23 ^b^
High fertility	40	10.6±1.38 a	5.85±0.69 ^ab^	2.56±0.45 ^b^	0.18±0.02 a	0.93±0.14 ^c^	24.8±3.59 ^c^
	50	10.7±2.41 a	6.86±0.49 a	2.58±0.25 ^b^	0.18±0.01 a	1.41±0.23 ^b^	34.8±3.88 ^b^
	60	10.5±0.49 a	6.45±1.22 a	3.51±0.41 a	0.21±0.06 a	1.92±0.15 a	45.1±3.24 a

a Values represent means ± SDs with 3 replicates (with each replicate composed of rice plant tissue sampled from different rhizoboxes of each treatment). Different letters in the same column indicate a significant difference at the *p*<0.05 level.

The rice biomass and N accumulation measurements differed significantly between the two fertility levels. The high-fertility soil increased the rice plant biomass by 90.4%, 65.2%, and 45.6% compared with the low-fertility soil when measured 40, 50, and 60 d after germination, respectively. The corresponding N accumulations increased by 47.9%, 48.6%, and 29.9% at the measurements taken 40, 50, and 60 d after germination, respectively (Table 2). High soil fertility markedly improved the growth and N uptake of the rice.

### Rice root morphological structure

We observed large changes in the morphological structure of the rice roots and found that the major characteristics of the developmental level of the root system such as root biomass, adventitious root number and root diameter were much greater in the high-fertility soil than in the low-fertility soil (Table 3). Furthermore, the characteristics of the rice root, such as POR, which indicated the developmental degree of aerenchyma, were significantly higher in the high-fertility soil than in the low-fertility soil (Table 3). In summary, the high-fertility soil markedly improved the growth of the rice root and the root porosity.

**Table 3:** Rice root growth and morphology^a^.

Fertility level	Sampling date (d)	Root biomass (g DW plant^−1^)	Adventitious root number (plant^−1^)	Root diameter (mm)	POR (%)
Low fertility	40	0.11±0.02^d^	22±3^d^	0.64±0.05^d^	14.1±1.46 ^e^
	50	0.20±0.03 ^c^	42±3 ^c^	0.78±0.06 ^c^	16.2±1.13^d^
	60	0.24±0.01 ^c^	49±3 ^c^	0.90±0.02 ^b^	16.9±1.38 ^cd^
High fertility	40	0.21±0.04 ^c^	28±3^d^	0.75±0.04 ^c^	19.6±1.44 ^bc^
	50	0.35±0.02 ^b^	61±13 ^b^	0.90±0.03 ^b^	22.0±2.92 ^ab^
	60	0.42±0.06 a	82±7 a	1.06±0.09 a	23.1±2.36 a

a Values represent means (means ± SDs), and different letters in the same column indicate a significant difference at the *p*<0.05 level. Data were pooled for n = 3, except for adventitious root diameter (n = 12), which was measured at the middle of each root selected for the average length of the whole root.

### Distribution of the soil oxygen concentration

**Figure 4:**
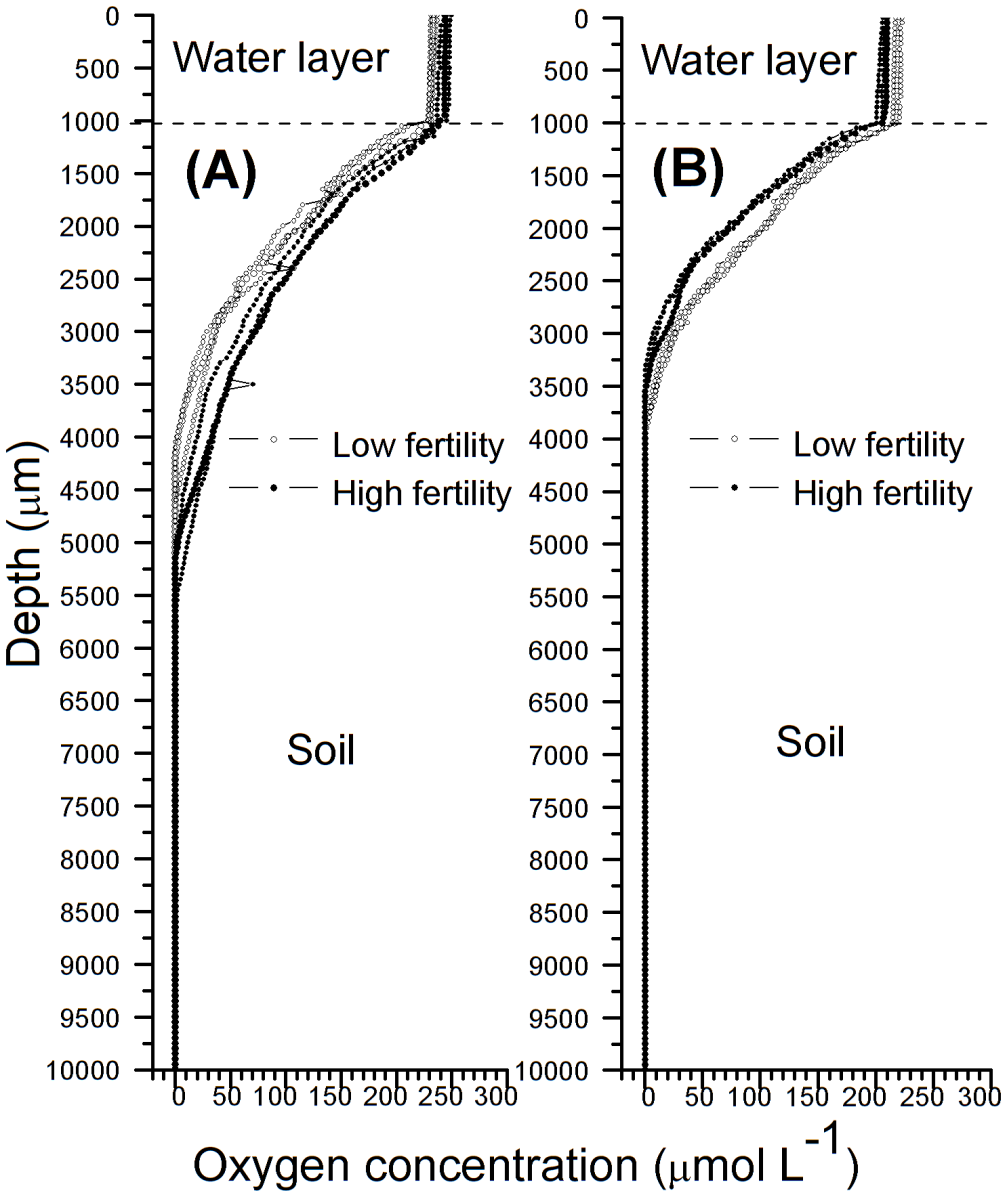
A representative graph showing oxygen profiles of (A) the rhizosphere soil (2 mm away from the root surface) and (B) the control (without rice plants) 50 d after germination in paddy soils with different fertility levels at 20°C. The oxygen microelectrode used was 20–30 µm in diameter (OXY25, Unisense, Aarhus, Denmark [31]). Three rhizoboxes for each treatment were used in the in situ measurements, and the oxygen concentration profile was determined in the right and left compartments of each rhizobox. All measurements were conducted in the centre of the range marked by a 2-mm distance from the nylon nets in the low- and high-fertility soils with and without the rice plant treatments. Hence, the measurements were repeated six times in each treatment. Three results (measured in the right compartment) are shown, and each curve shows one individual soil oxygen profile.

The oxygen dissolved in the water layer remained stable. The average values were approximately 231 and 243 µmol L^−1^ in the low-fertility and high-fertility treatments (n = 6; Fig. 4A), respectively, and 218 and 209 µmol L^−1^ in their respective controls (n = 6; Fig. 4B). Rice planting significantly increased the content of dissolved oxygen in the water layer. The oxygen concentration decreased with soil depth so rapidly that it became zero at a depth of approximately 2.6–4.2 mm below the soil surface (Fig. 4A, B). Interestingly, the oxygen concentration and oxygen penetration depth in the rhizosphere soil with high fertility were significantly higher than the corresponding values in the rhizosphere soil with low fertility, whereas a reverse trend existed in the controls of these two fertility levels, although certain values at certain depths showed no significant difference due to the presence of abnormal peaks. For example, at a depth of 2 mm from the soil surface, the average dissolved oxygen concentrations in the low- and high-fertility rhizosphere soils were 33.1 and 68.9 µmol L^−1^, respectively (n = 6; Fig. 4A), and their corresponding controls were 24.5 and 13.7 µmol L^−1^, respectively (n = 6; Fig. 4B). Among the six repetitions, the abnormal peaks appeared more often in the rice planting treatments than in the control. This finding may be a result of the rice root ROL.

### Root radial oxygen loss

**Figure 5:**
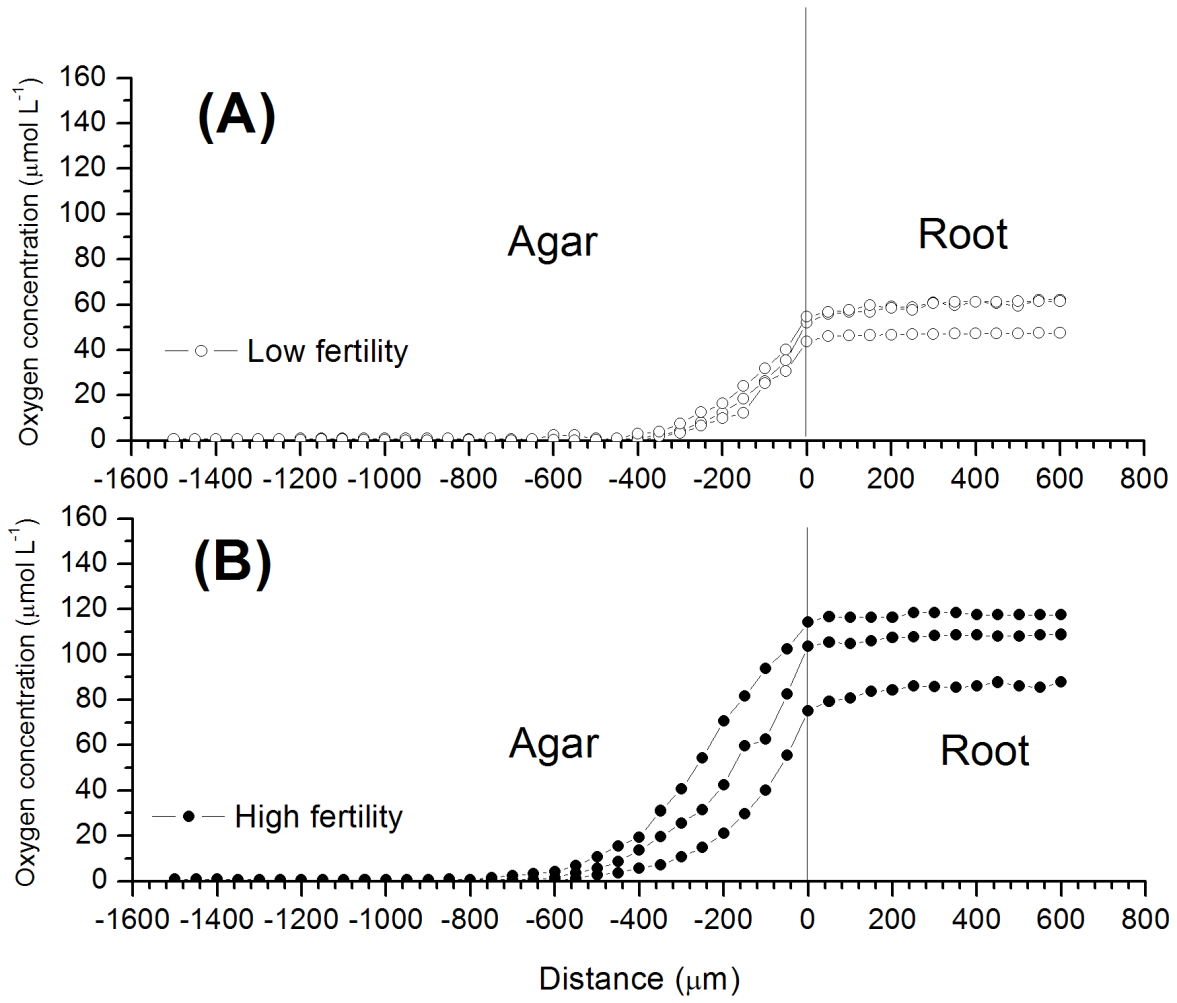
A representative graph showing oxygen profiles of the rhizosphere and within the rice root, 50 d after germination, growing in the (A) low- and (B) high-fertility soils at 20°C. The rice plant roots were separated from the soil and placed in agar with a nutrient solution. Newly formed roots with uniform 45-mm lengths were selected, and both were measured at a position 25 mm away from the root tips. The average diameters of the roots growing in the low- and high-fertility conditions were 0.74 and 0.93 mm at the measured points, respectively. The measurements were repeated in different rice plants and with different newly formed roots, with lengths ranging from 30 to 55 mm. An OXY10 oxygen microelectrode (Unisense, Aarhus, Denmark [31]) 8–10 µm in diameter was used to penetrate the rice root longitudinally. Three rhizoboxes were used for the individual rice root ROL measurements. Each curve shown in the figure indicates an individual rice root.

The oxygen in the agar was detected at an average distance of 400 µm from the root surface in the low-fertility treatment (Fig. 5A), in comparison with an average distance of 600 µm from the root surface in the high-fertility treatment (Fig. 5B). This finding indicated that the root ROL level of rice in the high-fertility soil was significantly higher than that in the low-fertility soil. The oxygen concentration decreased rapidly with distance near the rice root surface. When the microelectrode was in contact with the root surface, the average oxygen concentrations were 50.2 and 97.7 µmol L^−1^ in the low- and high-fertility treatments, respectively. The oxygen concentration remained unchanged inside the rice root, and the oxygen concentrations in the roots of the low-fertility treatment were lower than those in the high-fertility treatment (Fig. 5A, B). For example, the average oxygen concentrations in the low-and high-fertility treatments were 55.4 and 103.3 µmol L^−1^, respectively.

## Discussion

The penetration depths of oxygen in the soil surface and in the rhizosphere generally depend on both rice root ROL and the oxygen consumption caused by soil organisms and chemical oxidation [37]. In fact, rice planting could significantly alter (and, most likely, improve) the cycling of oxygen in the water and soil system due to rice root ROL, and our results confirmed this hypothesis (see Fig. 4A, B). For example, the dissolved oxygen concentration in the water layer was approximately 231 and 243 µmol L^−1^ in the low- and high-fertility treatments (Fig. 4A), respectively, compared with 218 and 209 µmol L^−1^ in their corresponding controls (Fig. 4B). In planted microcosms (planting with 110-d-old rice seedlings), oxygen is still detectable to a depth of at least 40 mm [38], which appears to be the maximum depth reported to date [23]. Interestingly, our results showed that the oxygen concentration in the rhizosphere soil with high fertility was significantly higher than that in the rhizosphere soil with low fertility (Fig. 4A), whereas the soil oxygen concentration in the high-fertility soil control was significantly lower than that in the low-fertility soil control (Fig. 4B). Because the control paddy soil without rice plants had no exogenous replenishment of oxygen as occurred for the rice root ROL, except for the dissolved oxygen provided by the flooding water, the high organic matter content in the high-fertility soil (see Materials and Methods) led to more severe oxygen depletion. This result may have been due to the higher oxygen consumption required by the degradation of organic matter and reduced compounds in the high-fertility soil compared with the low-fertility soil [37]. Although the oxygen content in the control high-fertility soil was lower than that in the control low-fertility soil (Fig. 4B), the soil oxygen concentration in the high-fertility paddy soil with rice plants was higher than that in the low-fertility soil (Fig. 4A), potentially due to the high rice root ROL in the high-fertility soil (Fig. 5B).

The difference in dissolved oxygen concentrations in rhizosphere soils between the high- and low-fertility soils appeared to be related to the differences in rice root ROL in these soils. When rice plants were grown in the high-fertility soil, the rice root growth was greatly improved; the root biomass, adventitious root number, and root diameter of the rice grown in the high-fertility soil were much greater than the corresponding values for the rice grown in the low-fertility soil (Table 3). A similar observation has previously been reported for wheat [39]. Not only did the rice root biomass, adventitious root number and root diameter change, but root porosity (see Table 3) was also improved by high soil fertility. This phenomenon does not appear to have been previously reported, as its positive feedback mechanism is unclear. ROL from rice roots estimated by Kludze *et al*. [19], showed a significant and positive correlation with POR. Oxygen efflux from root to soil represented 30%–40% of oxygen transportation from shoot to root [17]. Well-developed root systems and aerenchyma formation resulted in higher rice root ROL in the high-fertility soil (Fig. 5) [7] and consequently increased the oxygen content in the rhizosphere soil (Fig. 4A). Mugnai et al. [40] investigated maize roots and found that the root apex transition zone (intercalated between the apical division zone and the elongation zone [41]) plays central roles in both sensing and adapting to root hypoxia and emits the greatest amount of nitric oxide. This discovery motivated us to further investigate whether the root apex transition zone of rice plays a key role in altering the ROL and, correspondingly, the rhizosphere soil oxygen distribution.

An agar microcosm was used to further quantify the rice root ROL. The results indicated that the root ROL of rice planted in the high-fertility soil was greater than that in the low-fertility soil. The average oxygen concentrations at the root surface were 50.2 and 97.7 µmol L^−1^, and they decreased rapidly to nearly zero at distances of approximately 400 µm and 600 µm from the root surfaces in the low- and high-fertility treatments, respectively (Fig. 5A, B). The average oxygen concentrations in the interior of the rice roots in the low- and high-fertility treatments were approximately 55.4 and 103.3 µmol L^−1^, respectively (Fig. 5A, B). Similar results have been obtained by Revsbech *et al*. [23], who found that oxygen leakage created an oxic zone extending approximately 150 µm away from the fine lateral root (for 21-d-old rice); the inner oxygen concentration of the root (42-d-old rice) was approximately 75 µmol L^−1^. The rice root ROL can also promote the development of aerobic niches in the rhizosphere to restrict the accumulation of phytotoxic compounds (e.g., Fe^2+^, Mn^2+^, H_2_S) as well as denitrification [42] and maintain aerobic microbial processes, such as nitrification [14], [19].

Due to long-term fertiliser changes, soil fertility increased significantly and promoted soil nitrification, as widely reported in previous studies of paddy soil [8], [16] and upland soil [2], [43]. A low clay content contributed to a high soil porosity, which promoted gas transport by diffusion and advection, thereby supporting soil nitrification. The high ambient NH_4_^+^ pool in the high-fertility soil also improved its nitrification activity (Fig. 1 and 5). The maximal nitrification activities were found in the rhizosphere soil located 2 mm away from the root surface, and decreased with distance from the root surface (Fig. 1). In our previous study, the nitrification activity gradient also revealed nitrification activity differences in different rice cultivars. It also showed that the maximum nitrification activity detected in the rhizosphere soil, i.e., the maximum nitrification activities of the Indica- and Japonica- planted soils occurred 6 and 2 mm away from the rice roots, respectively [28]. The use of a rhizobox is convenient for distinguishing between the root surface and rhizosphere soil, but this design produces a dense root-zone and thus leads to a generally more oxidised environment, which may enhance nitrification. In contrast, more competition for NH_4_^+^ in this design may lower nitrification.

Based on our previous experience, a water layer of 1 cm that is maintained with applications of deionised water every morning and evening throughout the incubation would make it difficult to slice the soil. Additionally, mineral N, especially NO_3_^−^, would be dissolved in the overlying water because the oxygen concentration was much higher in the water layer than in the soil profile. If we continued to maintain a 1-cm water layer, the concentration of NO_3_^−^ in the soil would be very low and might even remain undetected because the paddy soil that we used is derived from Quaternary red clay and has relatively low pH values (usually 4–5.5). The nitrification activity in this paddy soil is much weaker than that in neutral paddy soil [26]. The NO_3_^−^ concentration in the paddy soil is correspondingly low. For this reason, we chose not to water on the evening of the day prior to the sampling date. The water layer was actually 1–2 mm deep when we measured the mineral N and nitrification activity. The advantage of this approach is that the soil is easy to slice, moreover, the NO_3_^−^ concentration can be measured more effectively. However, the disadvantage of this approach is that it imperceptibly stimulates nitrification and results in overestimates of the soil NO_3_^−^ concentration. In this experiment, the NO_3_^−^ concentration in the soil planted with rice did not differ significantly from that in the control. Because the nitrification activity in the rice planting soil was much higher than that in the control (Fig. 1D), the majority of the NO_3_^−^ resulting from increased nitrification might have been absorbed primarily by the rice roots. NO_3_^−^ can be absorbed rapidly by rice roots or can diffuse rapidly to other parts of the soil. Therefore, the NO_3_^-^ distribution in flooded paddy soil is relatively even. However, advection caused by evaporation from the leaves and following water transport to the roots increases transport processes in the soil to a greater extent than that resulting from diffusion. This pattern, was also confirmed by our previous experiment [28]. The low pH caused by acidic root exudates [44] and NH_4_^+^ (nitrification substrate) exhaustion in the root surface soil (Fig. 3A, B, and C) strongly inhibited root surface nitrification in both of the studied soil types (Fig. 1A, B, and C) [14]. Due to the high nitrification activity in the high-fertility soil, the NO_3_^−^ concentrations in the high-fertility soil were higher than those in the low-fertility soil (Fig. 2). This difference, might have caused more NO_3_^−^ uptake and assimilation by the rice plants grown in the high-fertility soil (the NRA results in Table 2 may support this hypothesis).

The NO_3_^−^ that is converted via nitrification in the root surface and rhizosphere soils is very important for N nutrition of rice [3], [4]. Extensive hydroponic experiments have demonstrated that rice growth, yield, net N acquisition, N translocation to the shoot, and NUE are greater with a mixed NH_4_^+^ and NO_3_^−^ supply than with NH_4_^+^ alone [3], [5], [6]. In the present study, the NO_3_^−^ concentration in the rice-planted soil was not significantly different from that measured in the control (Fig. 2), although the nitrification activity in the rice-planted soil was much higher than that in the control (Fig. 1), thus indicating that greater amounts of NO_3_^−^ absorbed by the rice root might have produced the results described above. NO_3_^−^ reductase is a key enzyme involved in NO_3_^−^ assimilation in crops, and its activity is strongly dependent on the external NO_3_^−^ concentration [28]. In this experiment, the rice leaf and root NRA in the high-fertility soil increased by 48.2% and 49.7%, respectively, compared with those in the low-fertility soil (Table 2), which also confirmed that more NO_3_^−^ was absorbed and assimilated by the rice planted in the high-fertility soil compared with that planted in the low-fertility soil. Greater amounts of NO_3_^−^ absorbed and assimilated by rice plants will also accelerate NH_4_^+^ uptake and assimilation, perhaps through the NO_3_^−^-specific induction of additional pathways for NH_4_^+^ assimilation [3], as also confirmed by the GSA results (Table 2). Hence, the rice N accumulation and rice growth of plants grown in the high-fertility soil were improved compared with those of plants grown in the low-fertility soil (Table 2).

## Conclusions

The present study investigated reasons that increasing soil fertility would enhance the growth of rice. The study adopted a new perspective focusing on rice root ROL and its corresponding soil oxygen profile. The fertile soil had high porosity (because of the low clay content) and a high NH_4_^+^ content, which supported soil nitrification. A high rhizosphere oxygen pressure distribution caused by the rice root ROL, which was attributed to the improved rice root growth and root porosity observed in the high-fertility soil, also contributed to the promotion of rhizosphere nitrification. The mechanism accelerating the development of rice root aerenchyma in the high-fertility soil requires further exploration. Investigation of the mechanisms of N loss and the quantification of N loss were not performed in this study but will be addressed by our laboratory in future research. Long-term tillage and fertilisation (especially organic fertiliser) would significantly increase soil fertility, but a large amount of organic matter would decrease the soil oxygen content. Fortunately, rice planting altered the water layer and soil oxygen status via root ROL, and rice in the fertile soil further improved the rhizosphere soil oxidisation environment. Our results suggest that increased fertilisation could lead to increased soil aeration in red soil regions in China, which might promote rice growth and rice N nutrition.
